# Building MOF Nanocomposites with Oxidized Graphitic Carbon Nitride Nanospheres: The Effect of Framework Geometry on the Structural Heterogeneity

**DOI:** 10.3390/molecules24244529

**Published:** 2019-12-11

**Authors:** Dimitrios A. Giannakoudakis, Teresa J. Bandosz

**Affiliations:** 1 Department of Chemistry and Biochemistry, The City College of New York, New York, NY 10031, USA; DAGchem@gmail.com; 2 Institute of Physical Chemistry, Polish Academy of Sciences, Kasprzaka 44/52, 01-224 Warsaw, Poland

**Keywords:** metal organic framework composites, oxidized graphitic carbon nitride nanoparticles, porosity, structural heterogeneity

## Abstract

Composite of two MOFs, copper-based Cu-BTC (HKUST-1) and zirconium-based Zr-BDC (UiO-66), with oxidized graphitic carbon nitride nanospheres were synthesized. For comparison, pure MOFs were also obtained. The surface features were analyzed using x-ray diffraction (XRD), sorption of nitrogen, thermal analysis, and scanning electron microscopy (SEM). The incorporation of oxidized g-C_3_N_4_ to the Cu-BTC framework caused the formation of a heterogeneous material of a hierarchical pores structure, but a decreased surface area when compared to that of the parent MOF. In the case of UiO-66, functionalized nanospheres were acting as seeds around which the crystals grew. Even though the MOF phases were detected in both materials, the porosity analysis indicated that in the case of Cu-BTC, a collapsed MOF/nonporous and amorphous matter was also present and the MOF phase was more defectous than that in the case of UiO-66. The results suggested different roles of oxidized g-C_3_N_4_ during the composite synthesis, depending on the MOF geometry. While spherical units of UiO-66 grew undisturbed around oxidized and spherical g-C_3_N_4_, octahedral Cu-BTC units experienced geometrical constraints, leading to more defects, a disturbed growth of the MOF phase, and to the formation of mesopores at the contacts between the spheres and MOF units. The differences in the amounts of CO_2_ adsorbed between the MOFs and the composites confirm the proposed role of oxidized g-C_3_N_4_ in the composite formation.

## 1. Introduction

Highly porous metal–organic frameworks (MOFs) are synthesized by the self-assembly of metal ions or clusters of them (as coordination centers) with polyatomic organic bridging linkages. In this process, 3D microporous structures are formed [1,2,3]. The diversity of the metal centers and organic ligands leads to materials of particular crystallographic structure, texture, and chemistry. Due to these properties, MOFs have been tested for various applications such as gas separation/storage [4,5,6,7,8,9], purification [10,11,12], sensing [13,14,15,16], electrodes for batteries [17,18], microextraction [19,20], detoxification of chemical warfare agents [21,22,23,24,25], and heterogeneous catalysis [26,27,28].

Even though MOFs can be considered as perfect porous materials of well-described geometry, this “perfection” has been recently found as limiting their performance, especially in separation and catalysis. In many of these applications, the hierarchical pore structure is needed and thus the homogeneity of the MOFs’ pore system, mainly related to micropores of specific sizes, can be disadvantageous for mass transfer processes. Moreover, uniformed chemistry, although advantageous for some applications, might limit the number of specific interactions/adsorption or catalytic centers. Therefore, the efforts have been intensified to introduce defects to the MOF structure targeting specific applications. Examples include mixed linkers [28,29,30], HCl treatment [31,32], variations in the synthesis conditions [33], the addition of molecular guests [34,35,36,37,38] or the incorporation of modified linkers [39,40]. These processes result in crystal imperfection, partial ligand replacement, or in nonbridging ligands, affecting the porosity, and the population, dispersion, and availability of active centers.

The composites of MOFs with graphite oxide (GO) showed an increased pore volume, conductivity, and chemical heterogeneity [41,42,43]. This trend was an outcome of the reaction of the copper centers of Cu-BTC and the O-containing (epoxy, carboxylic, hydroxyl, and sulfonic) or N-containing functional groups of the 2-D GO phase [42,43,44]. The oxygen groups of GO were suggested to act either as equatorial or axial linkers, replacing BTC or water molecules, respectively.

Since for building MOF-based composites, the geometry and morphology of the modifier is important, graphitic carbon nitride, g-C_3_N_4_, has also been used for this purpose. In its unoxidized form, it is an n-type semiconductor with a tunable band gap near 2.7 eV. g-C_3_N_4_ has a flake-like structure similar to that of graphite with mainly carbon and nitrogen organized in triazine and tri-s-triazine (or s-heptazine) units [45]. g-C_3_N_4_ was used to form composites with MIL-88A [46] to efficiently separate the photoinduced charge carriers. For its composites with Ti-based MOF [47] (MIL-125(Ti)), an enhanced photo-degradation of Rhodamine B was reported. For the synthesis process leading to true composites and not to physical mixtures, the interactions of a MOF phase and modifier functional groups are important. Thus, owing to these interactions, the composites of Cu-BTC and oxidized g-C_3_N_4_ had hierarchical porosity and exhibited photoactive properties [23].

Even though structural or chemical defects were not the focus of the synthesis procedure at the time of the introduction of MOF/other phase composites, the published results showed some distortion in the crystal structure, along with an increase in the porosity and in the population of metal centers [40,48]. Therefore, building the MOF composites with another phase can also be considered as a materials’ design strategy for introducing some defects to MOF crystals. Since these composites deserve another look at the origin of their surface activity, the objective of this paper was to present the comparison of the surface properties of the composites of two popular MOFs, HKUST-1 or Cu-BTC and UiO-66 with oxidized graphic carbon nitride nanospheres, with emphases on the formation of defects or/and new, physical/textural, optical, and chemical features. Since in both cases the same modifier is used, in the comparison presented, we focus on the geometry of MOF and its effects on the final properties of the composites.

## 2. Results and Discussion

**Figure 1:**
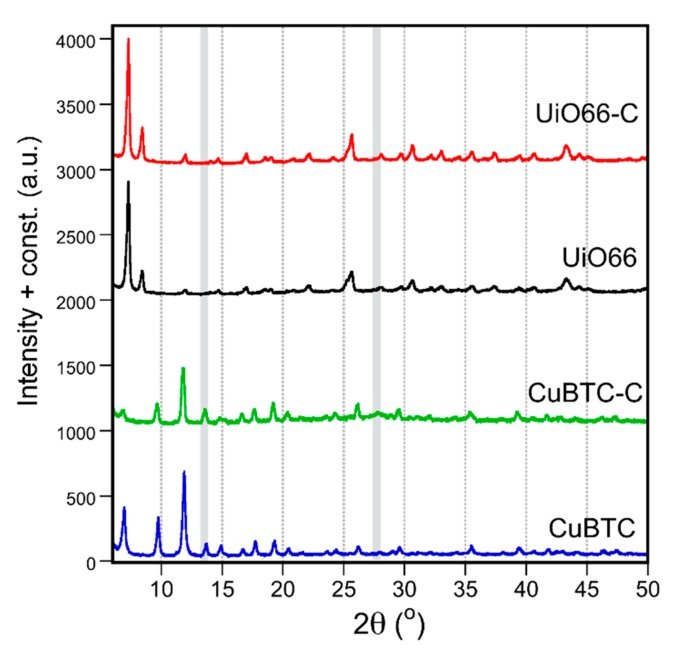
Comparison of x-ray diffraction patterns for MOFs and their composites.

The synthesized composites of oxidized g-C_3_N_4_ with Cu-BTC and UiO-66 are referred to as CuBTC-C and UiO66-C, respectively. They contain ~25% and ~10% of the oxidized g-C_3_N_4_ (gCNox) phase, respectively. In the evaluation of the outcomes of the synthesis of these materials, the analysis of the x-ray diffraction (XRD) patterns is important to assess the MOF structure features, formed in the presence of another phase. XRD patterns of the composites and their parent MOFs are presented in Figure 1. The patterns of Cu-BTC and UiO-66 follow those reported in the literature [49,50,51]. The preserved MOF structure was found in both composites. While in the case of CuBTC-C, the diffraction peaks were of a lower intensity than those for the parent MOF, the trend was the opposite in the case of UiO66-C. This suggests a different role of the modifier in the crystallization processes. The diffractogram of CuBTC-C indicates that the spherical nanoparticles of oxidized g-C_3_N_4_ with sizes of 10–50 nm [52] led to variations in the crystallization process, which caused minor changes in the lattice structure and morphology. The x-ray diffraction pattern of gCNox revealed two peaks at 27.6° and 13.5°, related to interplanar stacked graphitic layers [53]. For CuBTC-C, a broad and low intensity peak with a maximum at 26.7° was visible. The peak at 13.5° was not detected due to its overlap with an intense reflection of the framework. In the case of UiO66-C, where only 10% of the modifier was added, the absence of the peaks could be due to either the high dispersion of oxidized g-C_3_N_4_ or its small content.

**Figure 2:**
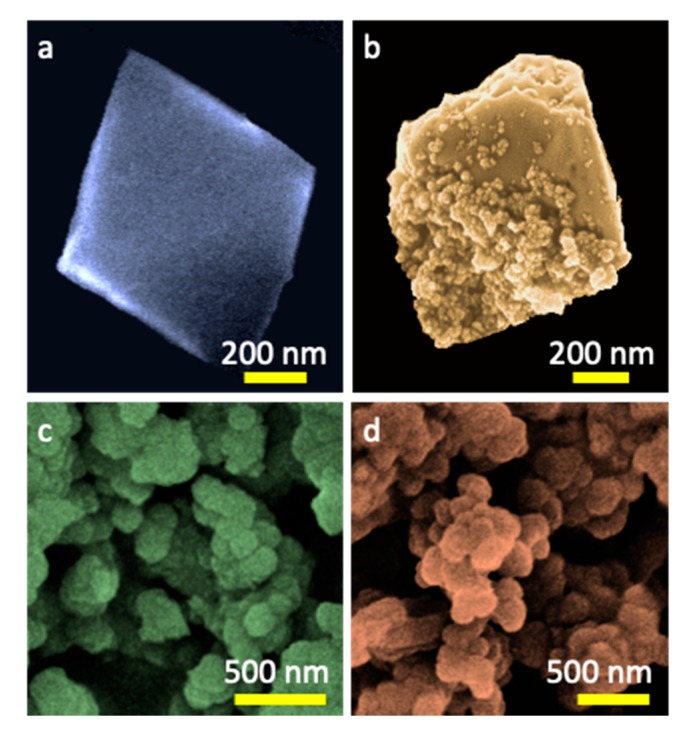
SEM images of CuBTC (**a**), CuBTC-C (**b**), UiO66 (**c**), and UiO66-C (**d**).

The morphology of the MOFs and their composites is compared in Figure 2. The CuBTC and CuBTC-C had octahedral shaped crystals, typical of this particular MOF. However, the crystals of the composite showed the visible effect of distortion demonstrated in their blunter edges and rough surfaces. That roughness was caused by the spherical nanoparticles, likely oxidized g-C_3_N_4_ [23], visible also on the crystals’ surfaces. In the case of UiO-66, the aggregates of semi-spherical particles with sizes between 90 to 190 nm were visible (Figure 2). For its composite, the aggregates were slightly smaller, and knowing that the sizes of oxidized g-C_3_N_4_ nanospheres are between 10–50 nm [23,52], it is not possible to determine the chemical homogeneity level of the material based only on the SEM images.

**Figure 3:**
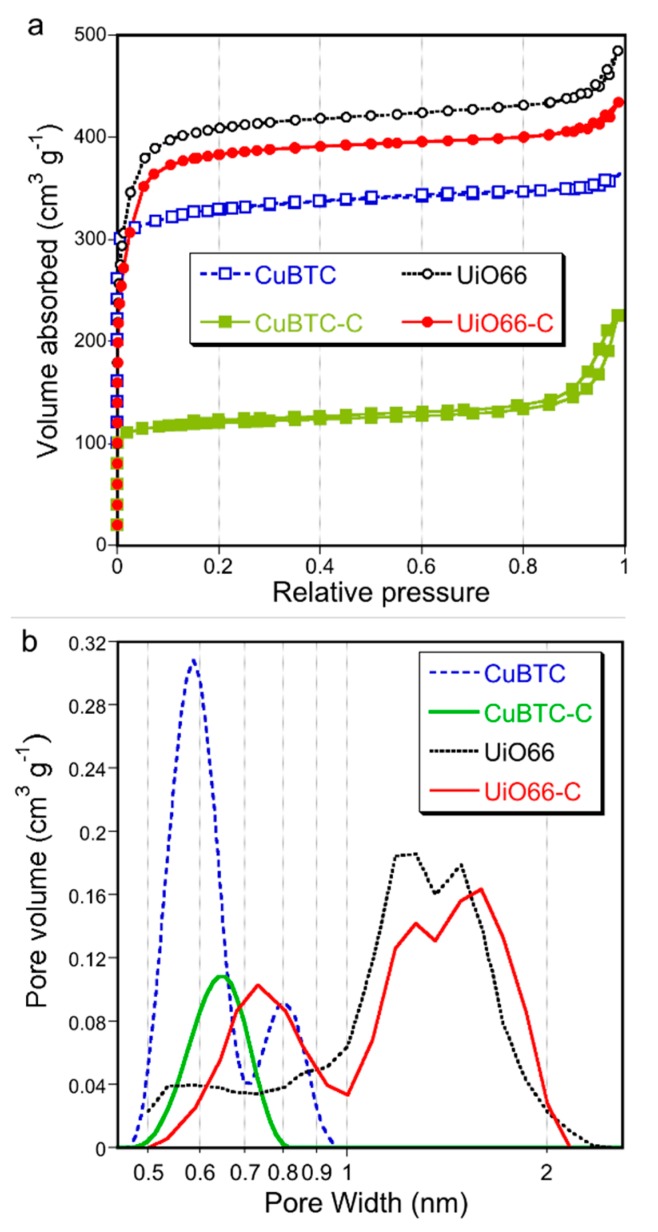
Nitrogen adsorption isotherms (**a**) and pore size distributions (**b**).

Since separation and catalysis are our target applications, the porosity of the synthesized materials was evaluated in detail from measured nitrogen adsorption isotherms (Figure 3a). The differences in the nitrogen uptake and in the shapes of the isotherms for the composites in comparison to those for pure MOFs are related to the alterations in the porous structure, upon the formation of the composites, especially for CuBTC-C. For this sample, the amount of nitrogen adsorbed decreased almost twice in comparison with that on CuBTC and the isotherm suggests the existence of mesopores. On the other hand, for UiO66-C, only small decreases in the amount adsorbed was seen in comparison to that on UiO66.

The pore size distributions (PSDs) were calculated from the isotherms using Non-Linear Density Functional Theory (NLDFT). Even though a specific kernel for this kind of material does not exist, the comparison of the results obtained for the same group of materials was considered as bringing meaningful information on the trend of textural alterations. The results suggest a more homogeneous distribution of micropores in CuBTC-C than that in CuBTC. The former sample also showed the presence of large pores with sizes between 5–50 nm (predominant 50 nm). The agreement of these pore sizes with the sizes of the oxidized g-C_3_N_4_ nanospheres suggests that these pores are a consequence of the incorporation of these nanoparticles inside the framework’s matrix. For UiO66-C, the formation of more pores with sizes between 0.7–1 nm (increase in their ratio to total pore volume) and the disappearance of small mesopores were the only visible changes in the PSD (Figure 3b).

**Figure 4:**
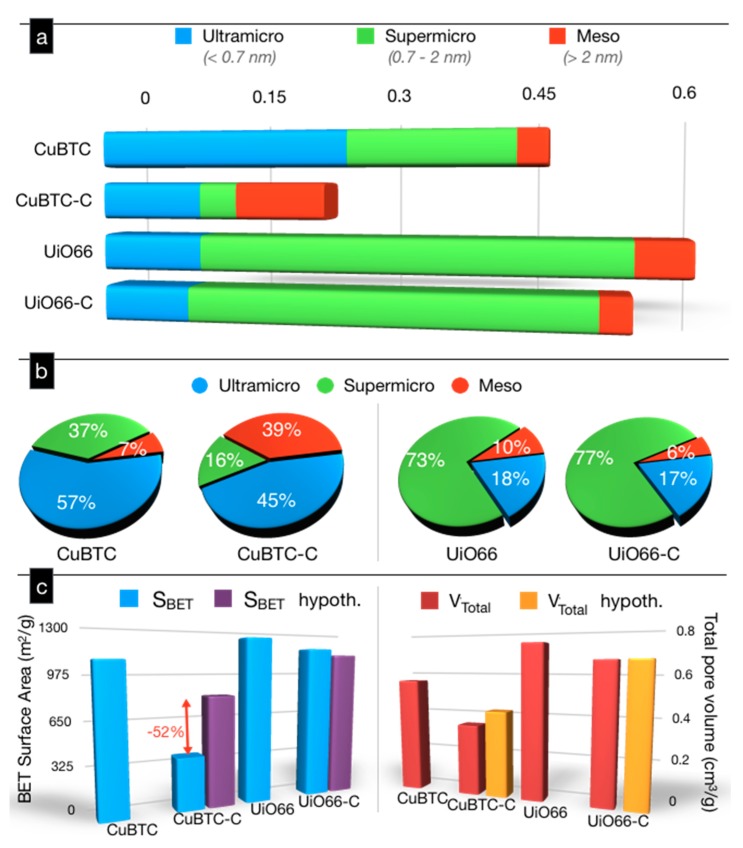
The comparison of the volumes of ultramicro-, supermicro-, and meso-pores (**a**), the percentages of each size range of pores (**b**), and a comparison of the measured and hypothetical (assuming physical mixtures) surface areas (S_BET_) and total pore volumes (V_Total_) (**c**).

The comparison of the pore volumes in the range of ultramicro-, supermicro-, and meso-pores for our samples is presented in Figure 4a. Figure 4b collects the percentages of the volumes in each range of the pore sizes per the total pores volume. In the case of CuBTC, the addition of the modifier led to a 50% decrease in the total pore volume. The volumes of the ultramicropores (<0.7 nm) and of the supermicropores (0.7–2 nm) decreased around 60% and 77%, respectively. That marked decrease in the volume of the supermicropores suggests that the nanospheres not only played a significant role in acting as linkers, but they also affected the crystallization/formation of the MOF phase and led to the formation of some amorphous or/and nonporous phases in the composite. Another plausible explanation of the decreased microporosity can be the blockage of the entrance of these pores by the gCNox nanoparticles. On the other hand, the volume of the mesopores in CuBTC-C increased three times when compared to that in CuBTC. The complex role of gCNox in the composite formation was also reflected in the ratio of ultramicro- to supermicro-pores (Figure 4b), which decreased from 0.65 for pure MOF, to 0.36 for the composite. For the UiO66 composite, the additive affected the structural features to a smaller extent and in a different way than in the case of Cu-BTC. The volumes of the ultramicro- and supermicro-pores decreased by 13 and 4%, respectively. The distribution of the PSDs indicated that the addition of nanospheres led to the formation of pores in the range of 0.6 to 0.9 nm. This, along with the same morphology of the composite as that of UiO66 (as seen on SEM images in Figure 2c,d), suggests that the nanospheres acted as nucleation centers, and the new pores were formed at the interface of the nanospheres and the MOF units. It is also interesting that the volume of the mesopores slightly decreased for this composite.

The extent of the effects of the same modifier addition on the alteration of the pore structure was also analyzed by comparing the measured surface areas and total pore volumes to those calculated for the hypothetical physical mixture (taking into consideration the contents of both phases and their specific contributions to porosity) (Figure 4c). For CuBTC-C, these parameters decreased 52% when compared to the physical mixture, indicating a marked effect of 25 wt.% oxidized g-C_3_N_4_ on the final porosity. Oxidized g-C_3_N_4_ is basically not very porous (surface area of 84 m^2^/g and the total pore volume of 0.482 cm^3^/g [52]) and its addition can contribute to the so called mass dilution effect in the physical mixture. The greater decrease of more than 25% supports a nonporous phase precipitation during composite synthesis and/or blocking of some microporosity of the MOF units by gCNox entities. On the other hand, the surface area of UiO66-C was 4% higher than that of the hypothetical physical mixture due to the formation of new pores, as discussed above.

**Figure 5:**
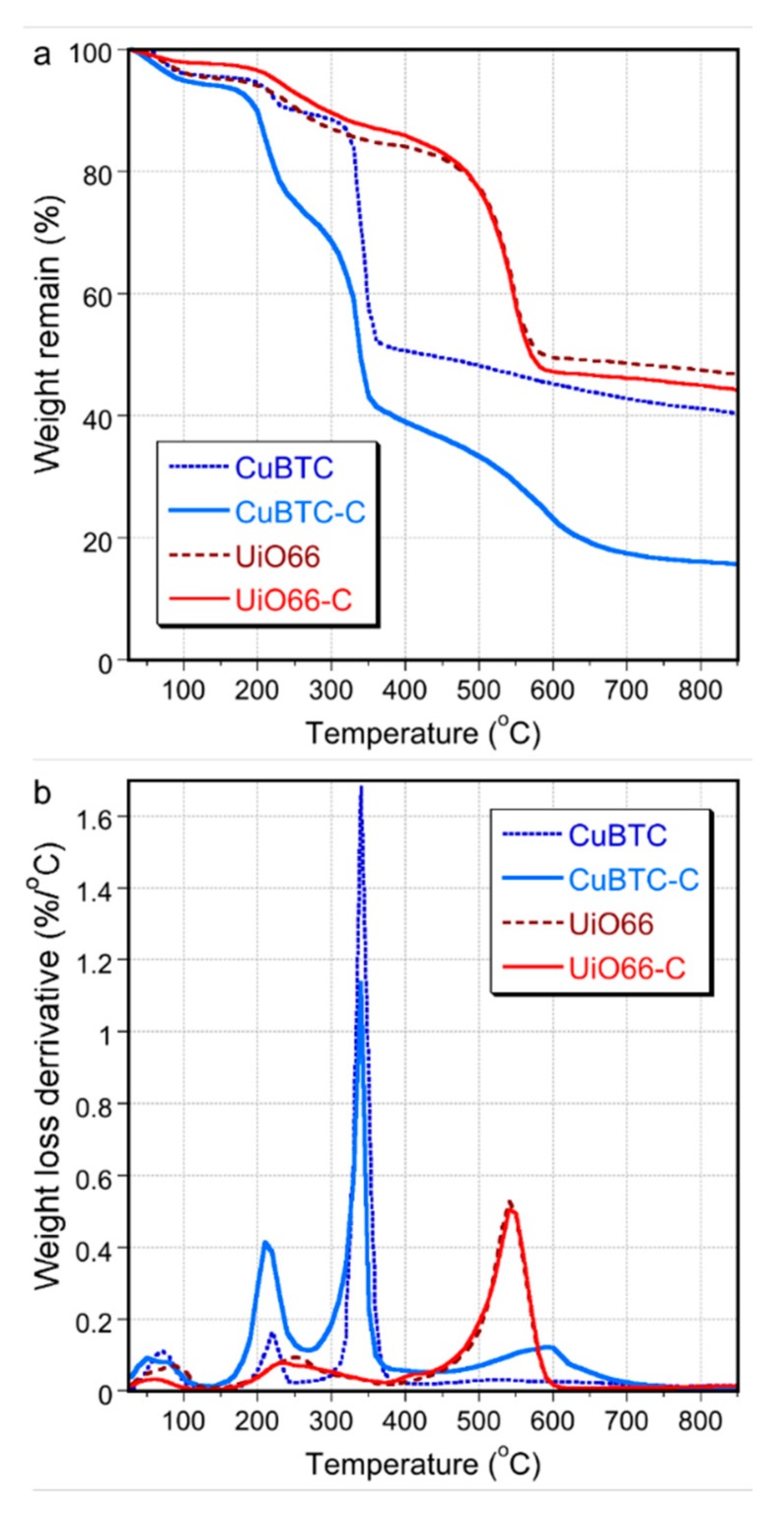
TG (**a**) and DTG (**b**) curves for the pure MOFs and their composites (measured in helium).

Thermal analysis experiments were performed in order to evaluate how the changes in the porous structure and chemistry affected the thermal stability of the composites. The thermogravimetric (TG) and derivative thermogravimetric (DTG) curves under a helium atmosphere are collected in Figure 5. It should be mentioned here that the weight loss of gCNox occurs continuously/gradually from room temperature up to complete combustion at 720 °C [23,52]. The thermal decomposition patterns of UiO-66 and UiO66-C are almost identical, suggesting limited chemical interactions of the MOF matrix with the nanospheres. The decomposition of the zirconium-based frameworks is visible as a peak at 520 °C revealed on the DTG curves for both samples. For the composite, the total weight loss was larger than that for the pure MOF due to the decomposition of the gCNox phase. The addition of the gCNox phase also led to a decrease in the affinity to retain water/decrease in hydrophilicity when compared to UiO66. In the case of CuBTC-C, the weight loss pattern revealed more pronounced differences in comparison to that for CuBTC, suggesting chemical heterogeneity and the involvement of nanospheres as linkers [54]. This is supported by the weight loss in the range from 160 to 260 °C, revealed only for the composite. The decomposition of CuBTC occurred between 310 and 370 °C and is seen as a peak on the DTG curve with a maximum at 340 °C. For the composite, the decomposition of the MOF phase started at a slightly higher temperature.

**Figure 6:**
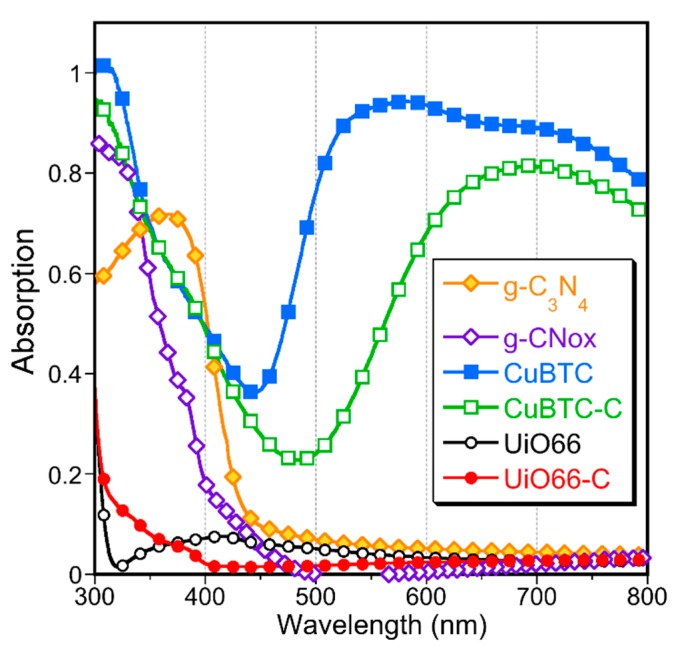
UV–Vis–NIR absorption spectra of the materials.

Since g-C_3_N_4_ is photoactive, its effect on the optical features of the composites was also evaluated. Defuse reflectance UV–Vis–IR spectra are collected in Figure 6. The coordination of the BTC ligands with the copper centers can occur in two planar symmetric bonding directions and in an axial direction [23,55]. For CuBTC-C, the latter coordination did not take place since its absorption spectrum did not show the characteristic absorption in the range from 450 to 530 nm [23]. The lack of this feature supports that the nanospheres acted as linkers and introduced a distortion of the ideal octahedral square grid due to π–π interactions with the BTC units [55]. Some alteration of the optical features was also observed in the case of UiO66-C. The broad absorption in the lower range of the visible range of light, revealed for UiO66, disappeared for the composite. For UiO66, absorption occurs in the ultraviolent range, up to 315 nm (~4 eV). Taddei and co-workers reported the band gap of this MOF as 4.1 eV (302 nm) [28] and showed that the defect engineering of UIO-66 based on modulated synthesis or post-synthetic linker exchange led to a decrease in the optical band gap. In the case of UiO66-C, the light absorption starting at 400 nm (3.1 eV) supports the decrease in the band gap compared to the pure UiO66.

**Figure 7:**
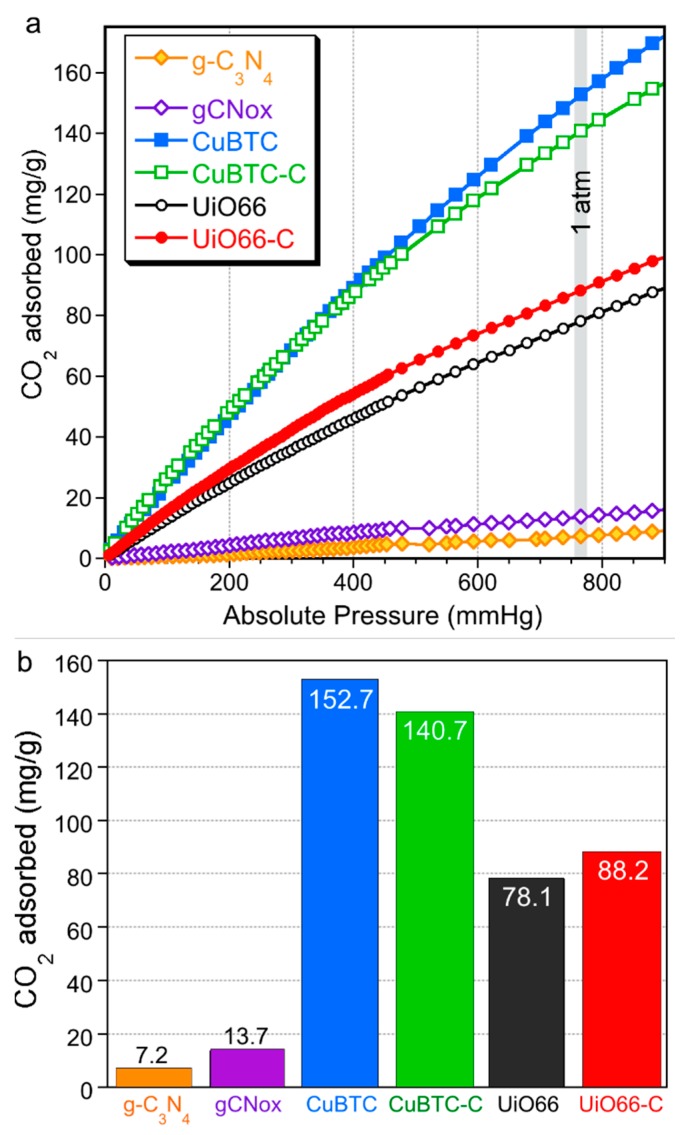
The CO_2_ adsorption isotherms (**a**) and the mg of CO_2_ adsorbed per gram of the materials at 1 atm (**b**).

The CO_2_ adsorption isotherms measured on our materials are presented in Figure 7a. The comparison of the amounts adsorbed at 1 atm and at 25 °C (expressed as mg/g) is included in Figure 7b. In the case of UiO-66-C, a 13% increase in the amount of CO_2_ adsorbed compared to that on MOF is linked to the formation of the modifier/MOF units’ interface providing small pores where CO_2_ could be adsorbed. UiO-66 is not expected to interact specifically with carbon dioxide molecules and Cao et al. presented a similar CO_2_ adsorption capability for UiO-66 [56] without the loss of adsorption even after five cycles. The CO_2_ adsorption results revealed an opposite trend in the case of copper frameworks, since the composite showed an 8% smaller uptake. Considering that the composite consisting of 25% gCNox adsorbed a limited amount of CO_2_, the addition of gCNox is beneficial for CO_2_ adsorption in the MOF phase. It is linked to the high level of defects in the latter and thus there is a higher availability of open copper centers for interaction with CO_2_ molecules [40,49]. On CuBTC-C and UiO66-C, 16% and 19% more CO_2_, respectively, is adsorbed than on the hypothetical mixtures of the components. The mechanisms of the CO_2_ adsorption on both MOFs support that in the case of CuBTC-C, mainly chemical/structural defects are responsible for the observed trend while those in UiO66-C are due to the development of small pores on the modifier/MOF unit interface. The comparison of the quantities of CO_2_ adsorbed on the materials tested are presented in Table 1. When the amount adsorbed is recalculated per units amount of the MOF phase, the amount adsorbed in the composites was about 25% higher than those on pure MOF. This effect is especially visible when the amount adsorbed per units of total pore volume of the adsorbent is compared. In such cases, CuBTC-C adsorbs 78% more CO_2_ than CuBTC.

**Figure 8:**
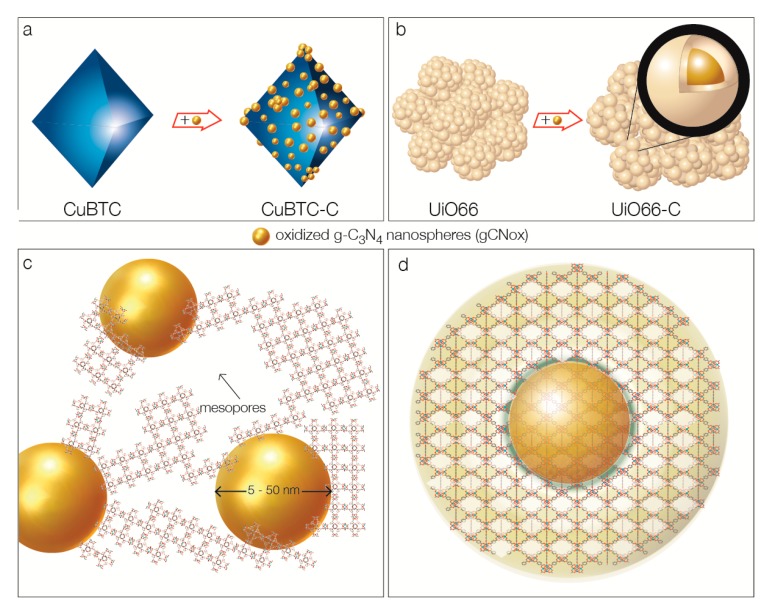
Visualization of the composites’ formation processes for CuBTC (**a**,**c**) and UiO66 (**b**,**d**).

The marked differences in the surface heterogeneity levels between the two composites with the same modifier but with different MOF (which also naturally must include the defects in the MOF structure in the broad sense of this word) are likely to be caused by the differences in the MOF structure geometry. While Cu-BTC is considered as having a simple cubic geometry, UiO-66 is more complex, both in its chemistry and the crystal structure. These differences might lead to the distinct levels of compatibility with the geometry and chemistry of the spherical modifier. It has been previously found that forming the composites of enhanced porosity such as those of MOF and 2-D graphite oxide (GO), besides the presence of functional groups that work as linkers [42,43,57,58], some compatibility of the MOF geometry and that of a modifier is required [57]. This is the case of CuBTC, whose units could align parallel to the flat surface of GO, leading to a significant increase in the porosity. The opposite effect was reported for MIL-125 (Ti-benzenedicarboxylate) [47], whose geometry prevented the porous composite formation [57]. Following this line of reasoning, the opposite effects are expected in the case of spherical modifiers. Thus, in the case of UiO-66, the nanospheres are considered as seeds around which, with the involvement of their functional groups, MOF crystals grow. This could explain a lack of clear distinction of oxidized g-C_3_N_4_ nanospheres in the SEM images and the small increase in the volume of ultramicropores of specific sizes. These pores likely represent the MOF/modifier interface. In the case of Cu-BTC, 25% of the geometrically incompatible spherical modifier not only did not contribute efficiently to the growth of the interface porosity/defects, but probably disturbed the yield of porous Cu-BTC units. The gCNox presence in the composite brought the mesoporosity formed between the units of MOF and modifier, but decreased the microporosity by hindering the MOF growing process. The visualization of these effects on the structure of the composites is presented in Figure 8.

## 3. Conclusions

The differences in the surface features of the composites of two distinctive MOFs, Cu-BTC (or HKUST-1), and UiO-66 (Zr-BDC), with the same modifier, the oxidized g-C_3_N_4_ nanospheres, indicate the importance of the geometrical compatibility between both composite constituents for the full utilization/development of interlayer space. Since the functional groups of the modifier are expected to work as linkers for the MOF units, those units have to be able to find the anchoring points/groups that will not be an obstacle to crystal growth, and this is apparently the case of the composite with the UiO-66. In the case of CuBTC, its crystals could not grow undisturbed on the spherical surface of the modifiers and this led to a significant obstruction in the MOF formation process. Nevertheless, some MOF units were formed, and they coexisted with the spheres of the modifier, resulting in the development of mesoporosity and hierarchical pore structure beneficial for mass transfer process. That disturbance in the MOF formation process led to the availability of more open metal sites, increasing CO_2_ adsorption on the composite per both unit mass and unit volume in the final materials.

Even though the detailed description of the defects formed in the composites is beyond the scope of this paper, we have shown that the composite formation by using nanoparticles as MOF indeed introduced structural and thus chemical surface heterogeneity that could enrich the application of these kinds of materials. We foresee that this approach can lead to tuning the structural, morphological, physico-chemical, or photochemical properties of the frameworks, bringing simultaneously new features of the unique MOF–modifier interfaces.

## 4. Experimental

### 4.1. Materials

The Hummers method was followed for the synthesis of oxidized graphitic carbon nitride nanoparticles (gCNox), starting with graphitic carbon nitride (g-C_3_N_4_) as the precursor [52,59]. The latter was obtained by the thermal treatment in air of dicyandiamide (Sigma-Aldrich) at 550 °C for 4 h in a horizontal furnace [60,61]. Details regarding the synthetic process of gCNox can be seen elsewhere [52].

The Cu-based MOFs were obtained following the synthetic protocol reported by Millward et al. [62]. For the composite, the targeted presentence of gCNox at the final material’s mass was 25 wt%. For the homogeneous dispersion/mixing, 5 min of mechanical stirring (600 rpm) and 30 min of sonication were performed after the addition of gCNox in the Cu-BTC precursor solutions. The remaining steps for the synthesis of the frameworks can be seen elsewhere [23,42]. The pure MOF is referred to as CuBTC and its composite with gCNox as CuBTC-C.

The Zr-based MOFs were obtained following a scaled-up synthetic process reported by Farha and Hupp with some minimal alterations [51]. In a glass reaction vessel were placed 30 mL of dimethylformamide (DMF), 6 mL of concentrated HCl, and 750 mg of ZrCl_4_. For complete dissolvement, 20 min of sonication was performed in an ultrasonication bath. The linker (terephthalic acid (BDC), 738 g) was dispersed in 20 mL DMF, and after 5 min of sonication, was inserted in the glass reaction vessel. The latter was sonicated for 20 min and afterward sealed hermetically and placed for 16 h in a furnace at 80 °C. After filtration and washing with DMF and ethanol, the received white powder was dried in a vacuum oven for 12 h (135 °C and 660 Torr) and the yield was found 83.2%. The composite was synthesized in the same way by adding 90 mg of gCNox (targeting a 10 wt% considering the yield) in together with the ZrCl_4_. The pure MOF is referred to as UiO66, while the composite is UiO66-C. The obtained dried powders were activated in high vacuum (10^−4^ Torr) at 150 °C (using ASAP 2020, Micromeritics) and were kept in hermetically closed vials prior the use [63].

### 4.2. Methods

The x-ray diffraction patterns were collected from 6 to 50 °C 2θ on a Philips Pert x-ray diffractometer (CuK_α_ radiation at 40 mA and 40 kV). A Zeiss Supra 55 VP microscope, equipped with a backscatter electron detector (acceleration voltage of 5 keV), was used to collect the SEM images. Nitrogen adsorption/desorption isotherms were measured at −196 °C on an ASAP 2020 (Micromeritic). The samples were outgassed at 120 °C for 16 h. From the nitrogen isotherms, the specific surface areas (S_BET_) were calculated using the Brunauer–Emmet–Teller method. The total pore volume (V_Total_) was evaluated based on the amount of nitrogen adsorbed at a relative pressure of ~0.99. The Non-Local Density Functional Theory (NLDFT) method was applied to calculate the pore size distributions (PSD) and the volume of ultramicropores (<0.7 nm), supermicropores (0.7–2 nm), and mesopores (>2 nm) [64,65,66]. Using the same DFT kernel for all samples allowed us to establish the trend in the PSDs. An SDT Q600 (TA instruments) thermal analyzer was used to measure the thermogravimetric (TG) curves from which derivative thermogravimetric (DTG) curves were obtained. The experiments were run in helium from room temperature to 1000 °C at a heating rate of 10 °C/min. Defuse reflectance (DR) UV–Vis–NIR spectroscopy was performed by using a spectrophotometer (Jasco V-570) equipped with an integrating sphere using Spectralon [poly(tetra-fluoroethylene)] as the baseline [67,68]. CO_2_ adsorption isotherms were measured using an ASAP 2020 (Micromeritics) under low pressure (0–900 mmHg). The experiments were performed at a constant temperature of 25 °C by immersing the tube inside a water bath in which water was circulated.

## Figures and Tables

**Table 1:** Comparison of quantities of CO_2_ adsorbed on the metal organic frameworks and their composites.

Quantity Adsorbed	CuBTC	CuBTC-C	UiO66	UiO66-C
mg/g (as in bars Figure)	152.7	140.7 (−8%)	78.1	88.2 (+13%)
mg/g of MOF phase	152.7	187.6 (+23%)	78.1	98.0 (+25%)
mg/cm^3^ of total pore volume	330	588 (+78%)	130	162 (+25%)
